# The relationship between smartphone use and dry eye disease: A systematic review with a narrative synthesis

**DOI:** 10.1097/MD.0000000000027311

**Published:** 2021-09-24

**Authors:** Khaloud Al-Marri, Maha Al-Qashoti, Hissa Al-Zoqari, Usra Elshaikh, Alya Naqadan, Raghad Saeed, Jameela Faraj, Mujahed Shraim

**Affiliations:** Department of Public Health, College of Health Sciences, QU Health, Qatar University, Doha, Qatar.

**Keywords:** dry eye disease, smartphone use, systematic review

## Abstract

**Background:**

Emerging research findings suggest a potential relationship between smartphone use (SPU) and dry eye disease (DED), which has not been systematically reviewed.

**Objectives:**

The aim of this study was to systematically review and synthesize evidence on the relationship between SPU and DED.

**Methods:**

A systematic search of Medline, EMBASE, CINAHL, and PsychINFO bibliographic databases from their inception to January 15, 2021. Study screening, full-text assessment, study selection and exclusion, data extraction, and quality assessment was performed independently by at least two review authors.

**Results:**

Four studies were included in the review (3 cross-sectional studies and 1 nonrandomized clinical trial). A narrative synthesis of findings was used due to heterogeneity among study designs and measures of association summarizing the relationship between SPU and DED. All included studies were conducted in South Korea and included school children, college students, or young adults. Three of the 4 included studies showed an association between SPU and DED.

**Conclusions:**

There is some evidence that SPU is associated with DED. However, this evidence is limited by a small number of studies of satisfactory methodological quality. There is a great need for high-quality studies to further investigate the relationship between SPU and DED and identify mechanisms underlying this potential relationship. This information is important for raising public awareness about the negative effect of SPU on eye health and development of clinical guidelines for this potentially emerging SPU-driven eye condition.

## Abstract 2

Supplemental Digital Content is available in the text

## 1 Introduction

Excessive smartphone use (SPU) or smartphone addiction is an emerging global public health problem. Smartphone addiction is defined as the excessive smartphone use, which is associated with functional impairment in activities of daily living and substance dependence-like-symptoms.^[1]^ A growing number of studies have shown that excessive SPU is associated with road traffic accidents and fatalities,^[2,3]^ higher stress scores,^[4]^ higher anxiety and depression scores,^[5–7]^ poor social relationships,^[8]^ sleep disturbance,^[9,10]^ low physical activity,^[7,11]^ fast food consumption and weight gain,^[11]^ and potentially dry eye disease (DED).^[12,13]^

The International Dry Eye WorkShop Study Group defines DED “…as a multifactorial disease of the tears and ocular surface that results in symptoms of discomfort, visual disturbance, and tear film instability with potential damage to the ocular surface. It is accompanied by increased osmolarity of the tear film and inflammation of the ocular surface.”^[14]^ The most common signs and symptoms of DED include eye fatigue, blurred or double vision, sore eyes, burning or stinging sensation, eye irritation and itching, and focusing problems.^[15]^ The common factors associated with DED include aging, female sex, Asian race, contact lens wear, environmental exposures (eg, low humidity and air pollution), use of visual display units (VDU), nutritional deficiencies (eg, Vitamin A deficiency), eye surgery, genetic factors, and some conditions, such as Sjogren syndrome, Meibomian gland dysfunction, diabetes, and somatoform disorders.^[16]^

The global prevalence of DED ranges between 5% and 50%.^[16]^ DED is associated with substantial economic burden. For example, although the prevalence of DED in the United States is relatively low (about 5%)^[17]^ as compared to other countries, 1 study showed that management of DED is associated with an annual average of $55 billion to the society of the United States.^[18]^ Additionally, signs and symptoms associated with DED, including discomfort and reduced vision quality, are associated with significant negative impact on mental health and quality of life due to difficulties in performing daily living activities.^[16,19]^ Research evidence shows that exposure to VDU such as computers and tables increases the risk of DED.^[15]^ Emerging epidemiological research using samples of schoolchildren and young adults suggests a potential relationship between SPU and DED.^[12,13,20,21]^ Establishing whether SPU is associated with DED has important implications for raising public awareness about the impact of SPU on eye health and development of clinical guidelines to minimize or prevent DED among smartphone users. To our knowledge, the relationship between SPU and DED has not yet been systematically reviewed. The aim of this systematic review was therefore to synthesize evidence on the relationship between SPU and DED.

## 2 Methods

### 2.1 Search strategy

Reporting of this systematic review was guided by the Preferred Reporting Items for Systematic Reviews and Meta-Analysis (PRISMA) statement (Supplemental file 1, http://links.lww.com/MD2/A469).^[22]^

Electronic searches of Medline, EMBASE, CINAHL and PsychINFO bibliographic databases, from their inception to January 15, 2021, were performed without any restrictions on publication language or study design. The search was conducted using controlled vocabularies (Medical Subject Headings (MeSH) or Emtree) and free text terms in all fields (all text) referring to SPU and DED (Supplemental file 2, http://links.lww.com/MD2/A470). The search terms included the following: (Smartphone (as MeSH or Emtree) OR smartphone OR “smart phone” OR cellphone OR “cell phone” OR “cellular phone” OR “mobile phone” OR “tablet phone”) AND (dry eye disease (as MeSH or Emtree) OR “dry eye“ OR ”keratoconjunctivitis sicca“ OR ”kerato conjunctivitis sicca“ OR ”keratitis Sicca“ OR ”corneal xerosis“ OR ”conjunctival xerosis“ OR ”meibomian gland dysfunction“ OR ”dysfunctional tear“ OR ”ocular dryness”). The reference lists of all relevant manuscripts were hand-searched to identify any additional relevant papers. In addition, citations of relevant articles were screened using the Web of Science Citation Index.

### 2.2 Criteria for considering studies for the review

#### 2.2.1 Types of studies

All epidemiologic study designs examining the relationship between SPU and DED were considered for inclusion. Due to lack of resources for translation, non-English language articles were excluded at full-text review stage and were reported as exclusions in the PRISMA flow diagram.

#### 2.2.2 Types of participants

We placed no limitations on type of participants in terms of age, gender, or any other sociodemographic characteristics. However, studies examining the relationship between SPU and DED in participants with any of the following common risk factors^[16]^ for DED were excluded at the full -text review stage: use of any eye drops, use of vitamin A therapy, current radiotherapy, oral contraceptive use and/or hormonal therapy, diabetes mellitus, facial palsy, atopic dermatitis, thyroid eye disease, and oophorectomy.

#### 2.2.3 Types of exposures

This review included studies of exposures involving daily duration of SPU in hours.

#### 2.2.4 Types of outcome measures

The outcome measure was DED measured using self-reported validated tools of DED and/or based on objective medical eye examination.

### 2.3 Study selection process

**Figure 1:**
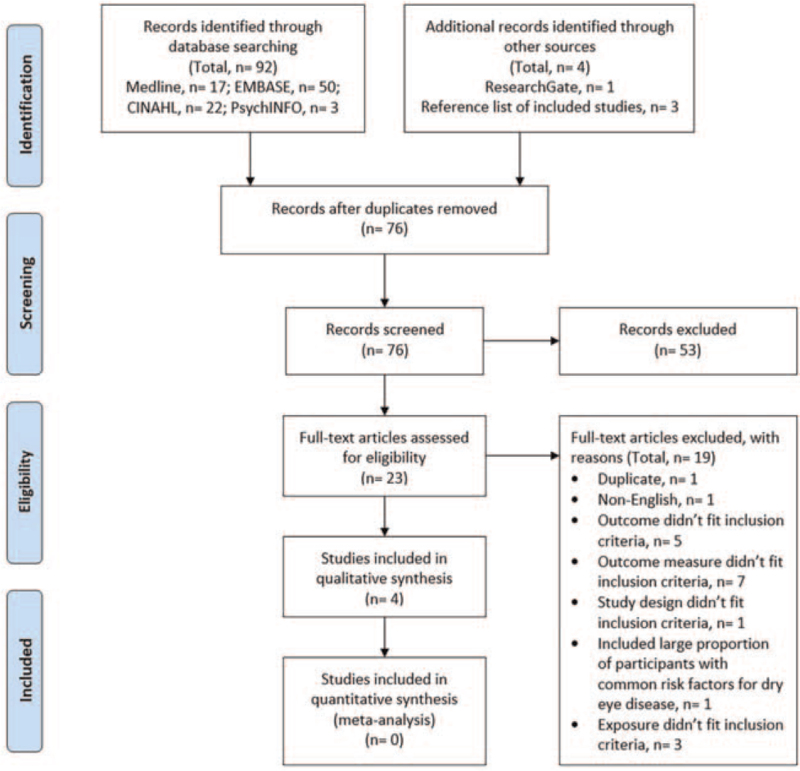
The PRISMA flow diagram of studies in the review.

Relevant studies meeting inclusion criteria were selected after two-stage reviewing process. In the first stage, duplicates were identified and removed, and irrelevant studies were excluded after screening their titles and abstracts independently by two reviewers using the Rayyan QCRI Web-based application.^[23]^ When final inclusion or exclusion decisions could not be made based on the titles and abstract, the full-text manuscripts were retrieved for a final decision at the second stage. In the second stage, 2 reviewers independently reviewed the full text of selected studies and made the final inclusion and exclusion decisions. Any disagreements between review authors were resolved by consensus or reconciled by a third review author (MS). The reasons for exclusion for excluded studies at the second stage were reported in the PRISMA flow diagram (Fig. 1).

### 2.4 Data collection and assessment of study quality

A standardized form was piloted and used to abstract the following information from each included study: main study characteristics (author name, publication year, country, study design and setting, sample size, demographic characteristics), data collection methods for assessment of SPU and DED, data analysis methods (univariable or multivariable analysis), and outcome measures of association between SPU and DED. The methodological quality was assessed and scored using an adapted scale for cross-sectional studies based on the Newcastle-Ottawa Assessment Scale for cohort studies (Supplemental file 3, http://links.lww.com/MD2/A471).^[24–26]^ The methodological quality of non-randomized studies was appraised using the methodological index for nonrandomized studies for comparative studies.^[27]^ Data abstraction and quality assessment of each included study were conducted independently by 2 review authors. Any disagreements were resolved by consensus or mediated by a third review author (MS).

## 3 Results

### 3.1 Study selection

A total of 92 citations were identified through the electronic search (Medline, n = 17; EMBASE n = 50; CINAHL, n = 22; PsychINFO, n = 3), 3 studies were identified from the reference lists of relevant studies, and 1 study was identified using ResearchGate. After removal of duplicates and screening of titles and abstracts, the manuscripts of 23 studies were retrieved for full-text assessment. Of those 23 studies, only 4 studies met the inclusion criteria and were included in the review. Figure 1 presents the study selection and exclusion process with reasons for exclusion at each stage of the process.

### 3.2 Study characteristics

The characteristics of included studies are shown in Table 1. All studies were conducted in South Korea and were published between 2014 and 2018. There were 3 cross-sectional studies^[12,13,20]^ and 1 nonrandomized clinical trial.^[21]^ Two studies included school children,^[12,13]^ 1 study included college students,^[20]^ and the remaining study included a community sample of young adults.^[21]^ The sample size ranged from 80 to 315 participants (total, n = 1599), with a proportion of females ranging from 37.5% and 73.5% (total females, n = 892; 55.8%). The mean age of participants ranged between 5.7 and 26.0 years.

**Table 1:** Characteristics of included studies.

Author, year	Country	Design	Population and setting	Sample size	Sex, % females	Age range (mean, SD)
Choi et al, 2018^[21]^	South Korea	NRT	Young adults, population based	80	37.5	21–36 (25.96, 2.98)
Choi et al, 2018^[20]^	South Korea	CS	College students	315	73.2	≥18 (20.82, 5.66)
Moon et al, 2014^[13]^	South Korea	CS	School children	288	50.7	11–12 (DED 11.00, 0.61; control 10.87, 0.66)
Moon et al, 2016^[12]^	South Korea	CS	School children	916	53.1	7–12 (9.90, 0.93)

CS = cross-sectional, DED = dry eye disease, NRT = non-randomized trial, SD = standard deviation.

### 3.3 Risk of bias within studies

Among the included studies, 2 studies^[13,21]^ were of good-quality and the remaining 2 studies were of satisfactory quality.^[12,20]^ The nonrandomized clinical study^[21]^ scored 18 points of 24 using the methodological index for non-randomized studies tool for not reporting on unbiased assessment of study endpoint and sample size calculation, and inadequate baseline equivalence of groups and statistical analysis (Table 2). The first cross-sectional study of good quality^[13]^ did not report about sample size calculation or describe characteristics of nonresponders and assessed SPU using a self-reported questionnaire (Table 3). The second cross-sectional study of satisfactory quality^[20]^ used a convenient sample of college students, provided no description of characteristics of nonresponders, and assessed SPU and DED using a self-reported questionnaire. The third cross-sectional study of satisfactory quality^[12]^ provided no description of representativeness of the sample, sample size calculation or characteristics of nonresponders, and assessed SPU using a self-reported questionnaire (Table 3).

**Table 2:** Detailed methodological index for nonrandomized studies for Choi et al, 2018 study^[21]^.

Study quality item	Not reported = 0	Reported but inadequate = 1	Reported and adequate = 2
1. A clearly stated aim			✓
2. Inclusion of consecutive patients			✓
3. Prospective collection of data			✓
4. Endpoints appropriate to the aim of the study			✓
5. Unbiased assessment of the study endpoint	✓		
6. Follow-up period appropriate to the aim of the study			✓
7. Loss to follow up less than 5%			✓
8. Prospective calculation of the study size	✓		
9. An adequate control group			✓
10. Contemporary groups			✓
11. Baseline equivalence of groups		✓	
12. Adequate statistical analyses		✓	
Total quality score = 18			

**Table 3:** Detailed Newcastle-Ottawa Scale of included cross-sectional studies.

	Selection				Comparability	Outcome		
Study	Representativeness of sample	Sample size	Nonrespondents	Ascertainment of exposure	Control for confounding factors	Assessment of outcome	Statistical test	Total quality score
Choi et al, 2018^[20]^	0	1	0	1	2	1	1	6
Moon et al, 2014^[13]^	1	0	0	1	2	2	1	7
Moon et al, 2016^[12]^	0	0	0	1	2	2	1	6

### 3.4 Results of individual studies

Owing to small number of included studies and between-study heterogeneity in study design and measures of association used between SPU and DED, a narrative synthesis of results was used. The first study used a nonrandomized clinical trial design to compare DED symptoms and markers between a “smartphone group” and a “computer display” control group. The smartphone group and the control group were assigned to play a puzzle game for 4 hours using a smartphone and a computer display, respectively. The DED symptoms and markers in both groups were measured at baseline and then after 1 hour and 4 hours of use. Using univariable analyses, this trial showed that the smartphone group had higher total ocular surface disease index (OSDI) score than the control group after 4 hours of smartphone use (mean = 25.03 SD = ±10.61 vs 6.61 ± 6.45, respectively, *P* <.05). No other measures of association or confidence intervals were reported. Similarly, the smartphone group had higher dichlorodihydro-fluorescein intensity values than the control group after 4 hours (141.56 ± 22.39 vs 123.03 ± 18.45, respectively, *P* < .5). These findings indicate higher dry eye symptoms severity in the smartphone group as compared to the control group. However, no statistically significant differences were observed between the 2 groups in other tear film function parameters and oxidation markers (Table 4).

**Table 4:** Summary of the relationship between smartphone use and dry eye disease.

Study	Exposure measurement	DED outcome measurement	Statistical analysis	Summary of association between SPU and DED
Choi et al, 2018^[21]^	Smartphone versus computer display use for 4 hours	OSDI, tear film function parameters, ROS parameters, and oxidation markers	Univariable	– Both groups had higher total OSDI scores at 4 h than baseline. However, the SPG had higher total OSDI score 25.03 ± 10.61 (mean ± SD) than the CG after 4 h 16.61 ± 6.45 (*P *< .5) – The SPG had lower TUBT and NIKBUT 4 h than baseline. However, there were no statistically significant differences between the SPG and the CG in TUBT (6.06 ± 1.92 vs 6.05 ± 1.73 s), NIKBUT (8.72 ± 4.79 vs 9.99 ± 5.46 s), Shirmer test (13.26 ± 3.21 vs 12.50 ± 2.59 mm), KEP score (0.30 ± 0.58 vs 0.45 ± 0.60), and TMH values (0.22 ± 0.08 vs 0.24 ± 0.12 mm) – The SPG had higher HEL values at 4 h than baseline. However, there were no statistically significant differences between the SPG and the CG in HEL (282.53 ± 14.08 vs 277.02 ± 54.04 nmol/L), 4.HNE (8.72 ± 4.79 vs 9.99 ± 5.46 μg/mL), MDA (13.26 ± 3.21 vs 12.50 ± 2.59 pmol/mg), and 8-OHdG (0.30 ± 0.58 vs 0.45 ± 0.60 ng/mL) – Both groups had higher total DCF fluoresceine intensity at 4 h than baseline. However, the SPG had higher DCF fluoresceine intensity than the CG after 4 h (141.56 ± 22.39 vs 123.03 ± 18.45) (*P* < .5)
Choi et al, 2018^[20]^	Self-reported daily duration of SPU in hours	OSDI	Multivariable	Increase in SPU duration in hours was associated with higher mean OSDI scores (1–2 h: 22.34 ± 16.12, 2–3 h: 22.69 ± 17.09, 3–5 h: 30.76 ± 19.80, ≥5 h: 31.32 ± 20.13 (F = 5.133, *P* = .002). However, SPU was not associated with OSDI mean scores in multivariable analysis (data was not reported)
Moon et al, 2014^[13]^	Self-reported daily duration of SPU in hours	Dry Eye Disease Diagnostic Criteria of the International Dry Eye WorkShop 2007	Multivariable	Increase in daily SPU duration by 1 h was associated with increased odds of DED (OR = 1.86, 95% CI 1.07, 3.24)
Moon et al, 2016^[12]^	Self-reported daily duration of SPU in hours	Dry Eye Disease Diagnostic Criteria of the International Dry Eye WorkShop 2007	Multivariable	– Increase in daily SPU duration by 1 h was associated with increased odds of DED (OR = 13.07, 95% CI 5.99–28.52) – DED rate in participants diagnosed with DED who stopped SPU for 4 wk decreased by 100% as compared to 13.3% in those diagnosed with DED but who continued SPU for 4 wk

4-HNE = 4-hydroxy-2-nonenal, 8-OHdG = 8-oxo-2’-deoxyguanosine, CG = control group, CI = confidence interval, DCF = dichlorodihydro-fluorescein, DED = dry eye disease, HEL = hexanoyl lysine, KEP = keratoepitheliopathy, MDA = malondialdehyde, NIKBUT = non-invasive keratograph break up time, OSDI = ocular surface disease, ROS = reactive oxygen species, SD = standard deviation, SPG = smartphone group, SPU = smartphone use, TBUT = tear break up time, TMH = tear meniscus height.

The second study examined the association between daily SPU duration in hours and OSDI scores using a self-reported cross-sectional questionnaire.^[20]^ Using univariable analysis, this study found that increase in SPU duration in hours was associated with higher mean OSDI scores (1–2 hours.: 22.34 ± 16.12 [values are mean ± standard deviation], 2–3 hours.: 22.69 ± 17.09, 3–5 hours.: 30.76 ± 19.80, ≥5 hours: 31.32 ± 20.13 [F = 5.133, *P* = .002]). However, in multivariable analysis, SPU was not associated with OSDI score (data was not reported) (Table 4).

The third study examined the relationship between SPU in hours measured using a self-reported cross-sectional questionnaire and DED measured based on both self-reported dry eye symptoms and clinical eye examination.^[13]^ DED was defined and diagnosed according to the International Dry Eye WorkShop 2007.^[14]^ This study showed that increase in daily duration of SPU by one hour was associated with increased odds of DED by 1.86 times (95% confidence interval 1.07–3.24).

The fourth study^[12]^ assessed the association between daily duration of SPU in hours and DED based on the International Dry Eye WorkShop 2007.^[14]^ The authors of that study referred to it as a case-control study, but the design was an analytical cross-sectional study. Additionally, participants diagnosed with DED in this study were instructed to stop SPU for 4 weeks to explore SPU cessation effect on DED symptoms and severity. This study reported that an increase in daily duration of SPU by 1 hour was associated with higher odds of DED by 13.07 times, (95% confidence interval 5.99–28.52). In addition, DED in children who stopped SPU over a 4-week duration had reduction in DED rate by 100% as compared to 13.3% among children with DED who continued SPU for 4 weeks (*P* < .001) (Table 4).

## 4 Discussion

### 4.1 Summary of main findings

This systematic review was conducted to investigate the relationship between SPU and DED. Only four studies met the inclusion criteria for this systematic review. Because of methodological differences and limitations of included studies, we could not pool their findings using meta-analysis. Overall, the findings of three studies were consistent and indicated that SPU is associated with DED. However, due to methodological limitations and high risk of selection and information bias in included studies, the findings of this systematic review should be interpreted with caution.

### 4.2 Comparison with existing literature

This is the first systematic review to synthesize the evidence base about the relationship between SPU and DED. The findings from this review are consistent with findings of a previous systematic review indicating that SPU was associated with eye eyestrain signs and symptoms.^[28]^ The mechanisms underlying the observed association between SPU and DED is not very clear. However, a limited evidence suggests that the association between SPU and DED could be explained by reduced blink rate, incomplete blink, reduced tear volume and reduced tear break-up time due to holding the smartphone beneath the eye level (lower gaze angle) and constant cognitive attention.^[15]^ For example, one of the studies included in the present systematic review showed that cessation of SPU was associated with improvement in tear break-up time among school children.^[12]^

### 4.3 Strengths and limitations

This is the first systematic review to examine the relationship between SPU and DED. The review used a rigorous methodology and was reported based on the PRISMA statement. To identify relevant studies, we used a comprehensive search covering multiple key bibliographic databases. In addition, selection of relevant studies, data extraction, and assessment of study quality was conducted independently by at least two review authors and by following a clear study inclusion criteria and validated quality assessment scales. However, this review has some limitations. First, all included studies had important methodological limitations (such as lack of priori sample size calculation, convenient sampling, and inadequate control for confounding) and high risk of selection and information biases. Three of the included studies measured SPU using self-reported subjective data, which may be subject to recall or reporting biases. In addition, 2 studies relied on subjective self-reported and DED symptoms without an objective clinical eye examination. Second, 3 studies used a cross-sectional design, and therefore, the direction of association between SPU and DED cannot be established. Third, although our systematic search was not restricted by language of publication of primary studies, one relevant study published in Korean was excluded at the full-text review stage. However, no studies were excluded based on their methodological quality. Fourth, our search did not systematically cover the grey literature. However, we hand-searched the reference lists of relevant studies and screened their citations to identify any more relevant studies. Fifth, all included studies were conducted in South Korea, and therefore, the findings of this review may not necessarily generalize to other populations from other countries or regions. Sixth, the current review protocol was not prospectively registered. However, no deviations from the original review protocol occurred.

### 4.4 Implications for practice and future research

Despite the satisfactory methodological quality of included studies, the present systematic review found an association between SPU and DED, which accords with existing knowledge about a positive association between VDU use, such as computers, and DED. These findings have important implications for clinical practice such as raising public awareness about the negative impact of SPU on eye health and development of clinical guidelines to minimize DED symptoms severity or prevent DED among smartphone users. There are a limited number of primary studies examining the association between SPU and DED. Given the high SPU penetration rate in the community, high quality research studies are needed to further investigate the association between SPU and DED, assess whether SPU increases dry eye symptoms severity among individuals with DED, and uncover the exact mechanisms underlying this association. In addition to subjective measures of DED symptoms, future research should use validated and objective measures of SPU and DED. Moreover, future studies should use well-controlled research methods, such as adequate sample size, blind assessment of SPU and DED, and adequate control for confounding.

## 5 Conclusions

There is limited evidence suggesting a relationship between SPU and DED. There is a great need for high-quality studies to further investigate the relationship between SPU and DED and identify mechanisms underlying this potential relationship. This information is important for raising public awareness about the negative effect of SPU on eye health and development of clinical guidelines for this potentially emerging SPU-driven eye condition.

## Acknowledgment

The authors thank Professor Lukman Thalib for taking part in study conceptualization.

## Author contributions

**Conceptualization:** Shraim Mujahed.

**Data curation:** Khaloud Al-Marri, Maha Al-Qashoti, Hissa Al-Zoqari, Usra Elshaikh, Alya Naqadan, Raghad Saeed, Jameela Faraj, Mujahed Shraim.

**Funding acquisition:** Mujahed Shraim.

**Investigation:** Mujahed Shraim.

**Methodology:** Mujahed Shraim.

**Project administration:** Mujahed Shraim.

**Supervision:** Mujahed Shraim.

**Writing – original draft:** Khaloud Al-Marri, Maha Al-Qashoti, Hissa Al-Zoqari, Usra Elshaikh, Alya Naqadan, Raghad Saeed, Jameela Faraj, Mujahed Shraim.

**Writing – review & editing:** Khaloud Al-Marri, Maha Al-Qashoti, Hissa Al-Zoqari, Usra Elshaikh, Alya Naqadan, Raghad Saeed, Jameela Faraj, Mujahed Shraim.

## Supplementary Material

SUPPLEMENTARY MATERIAL
