# Supplementary material for: The relationship between smartphone use and dry eye disease: A systematic review with a narrative synthesis
Source: Medicine (Baltimore). 2021 Sep 24;100(38):e27311. doi: 10.1097/MD.0000000000027311 (PMC10545216; doi:10.1097/MD.0000000000027311)
Supplement: SUPPLEMENTARY MATERIAL [file medi-100-e27311-s002.docx]

**Supplemental file 2** Detailed search strategy used to identify relevant studies for the systematic review

| **Concept** | **Medline** | **EMBASE** | **CINAHL** | **PsychINFO** |
| --- | --- | --- | --- | --- |
| Smartphone | Smartphone/  Smartphone  “Smart phone”  Cellphone  “Cell phone”  “Cellular phone”  “Mobile phone”  “Tablet phone” | Smartphone/  Smartphone  “Smart phone”  Cellphone  “Cell phone”  “Cellular phone”  “Mobile phone”  “Tablet phone” | Smartphone/  Smartphone  “Smart phone”  Cellphone  “Cell phone”  “Cellular phone”  “Mobile phone”  “Tablet phone” | Smartphone use/  Smartphone  “Smart phone”  Cellphone  “Cell phone”  “Cellular phone”  “Mobile phone”  “Tablet phone” |
| Dry Eye Disease | Dry Eye Syndromes/  “Dry eye"  "Keratoconjunctivitis Sicca"  "Kerato conjunctivitis Sicca"  "Keratitis Sicca"  "corneal xerosis"  "conjunctival xerosis"  "Meibomian Gland Dysfunction"  "Dysfunctional tear"  "Ocular dryness" | Dry Eye/  “Dry eye"  "Keratoconjunctivitis Sicca"  "Kerato conjunctivitis Sicca"  "Keratitis Sicca"  "corneal xerosis"  "conjunctival xerosis"  "Meibomian Gland Dysfunction"  "Dysfunctional tear"  "Ocular dryness | Dry Eye Syndromes/  “Dry eye"  "Keratoconjunctivitis Sicca"  "Kerato conjunctivitis Sicca"  "Keratitis Sicca"  "corneal xerosis"  "conjunctival xerosis"  "Meibomian Gland Dysfunction"  "Dysfunctional tear"  "Ocular dryness" | Eye Disorders/  "Dry eye"  "Keratoconjunctivitis Sicca"  "Kerato conjunctivitis Sicca"  "Keratitis Sicca"  "corneal xerosis"  "conjunctival xerosis"  "Meibomian Gland Dysfunction"  "Dysfunctional tear"  "Ocular dryness" |
