# Supplementary material for: The relationship between smartphone use and dry eye disease: A systematic review with a narrative synthesis
Source: Medicine (Baltimore). 2021 Sep 24;100(38):e27311. doi: 10.1097/MD.0000000000027311 (PMC10545216; doi:10.1097/MD.0000000000027311)
Supplement: SUPPLEMENTARY MATERIAL [file medi-100-e27311-s003.docx]

**Supplemental file 3** Newcastle - Ottawa Quality Assessment Scale (adapted for cross-sectional studies)

**Selection:** (Maximum 3 stars)

1) Representativeness of the sample:

a) Truly representative of the average in the target population. * (all subjects or random sampling)

b) Somewhat representative of the average in the target population. * (non-random sampling)

c) Selected group of users.

d) No description of the sampling strategy.

2) Non-respondents

a) Comparability between respondents and non-respondents characteristics is established, and the response rate is satisfactory. *

b) The response rate is unsatisfactory, or the comparability between respondents and non-respondents is unsatisfactory.

c) No description of the response rate or the characteristics of the responders and the non-responders.

3) Ascertainment of the exposure (risk factor)

a) Validated measurement tool. *

b) Non-validated measurement tool, but the tool is available or described.

c) No description of the measurement tool.

**Comparability:** (Maximum 2 stars)

1) The subjects in different outcome groups are comparable, based on the study design or analysis. Confounding factors are controlled.

a) The study controls for the most important factor (select one). *

b) The study control for any additional factor. *

**Outcome:** (Maximum 2 stars)

1) Assessment of the outcome

a) Independent blind assessment. *

b) Record linkage. *

c) Self report.

d) No description.

2) Statistical test

a) The statistical test used to analyze the data is clearly described and appropriate, and the measurement of the association is presented, including confidence intervals and the probability level (p value). *

b) The statistical test is not appropriate, not described or incomplete.

Adapted from:

- Ouzzani M, Hammady H, Fedorowicz Z, Elmagarmid A. Rayyan—a web and mobile app for systematic reviews. Systematic Reviews. 2016;5(1):210.
- Wells G, Shea B, O Connell DL, et al. The Newcastle-Ottawa Scale (NOS) for Assessing the Quality of Nonrandomised Studies in Meta-Analyses. 2014.
